# A new sexual revolution

**DOI:** 10.1590/S1679-45082014ED3182

**Published:** 2014

**Authors:** Carmita Helena Najjar Abdo

**Affiliations:** * Program of Studies in Sexuality (ProSex), Faculdade de Medicina, Universidade de São Paulo, São Paulo, SP, Brazil

For the fourth edition of the Diagnostic and Statistical Manual of Mental Disorders (DSM-IV),^(1)^ sexual dysfunctions are defined by the presence of pain or an alteration during one or more of the phases of the linear sexual response cycle (desire – arousal – orgasm – resolution).

In the fifth edition of this manual, DSM-5,^(2)^ published in 2013, sexual dysfunctions were considered gender-specific, in which the difficulties with desire and arousal of the woman constitute a single dysfunction (female sexual interest/arousal disorder). The genito-pelvic pain/penetration disorder is also now one single category, replacing, respectively, dyspareunia and vaginismus (as they were in DSM-IV), since according to North-American specialists, they are conditions difficult to distinguish.

As to subtypes, the dysfunctions continue to be characterized as lifelong (primary) or acquired (secondary), and generalized (when they occur under any circumstance) or situational. But the intensity of distress (mild, moderate, or severe) now also composes the characterization of the dysfunctions and the context should be specified: factors relative to the partner, to the relationship, to individual vulnerability, to culture, to religion, and those of medical origin must be reported.

A minimum duration of six months has been established as a criterion for the diagnosis of sexual dysfunction in order to avoid “overdiagnosis” and to increase precision, distinguishing transient sexual difficulties from more persistent sexual dysfunction, and therefore, pathological problem.

As to issues related to gender incongruence, united as gender identity disorder in DSM-I V, a new (less stigmatizing) diagnostic denomination has been suggested by DSM-5: gender dysphoria, considered a multicategory concept, since it encompasses gender dysphoria in children, adolescents, and adults.

The previous criterion A (cross-gender identification) and B (aversion towards one's gender) have been merged, as there was no evidence that could justify keeping the two separate.

In children, “a strong desire to be of the other gender” substitutes the previous “repeatedly stated desire” (from DSM-IV) to encompass the condition of some children who, within a coercive environment, may not verbalize this desire. The criterion A1 for children, “a strong desire to be of the other gender or an insistence that he or she is the other gender” is necessary, but not sufficient, for the diagnosis, which is now more restrictive.

An ample change was proposed for the paraphilias in DSM-5: paraphilia is not, in and of itself, a mental disorder, and should be distinguished from paraphilic disorder. Paraphilia becomes a disorder when it causes distress or impairment to the individual or a paraphilia whose satisfaction has entailed personal harm or risk of harm to others. Therefore, paraphilia is a necessary but not sufficient condition for the diagnosis of paraphilic disorder and consequently, does not require automatic clinical intervention.

The distinction between paraphilia and paraphilic disorder did not change the diagnostic definitions already established since DSM-III-R.^(3)^ In DSM-5, two criteria stand out: criterion A, which specifies the nature of paraphilic arousal (with woman's underwear, for example, such as in fetishism) and criterion B, which points to the negative consequences (distress, impairment in other areas of life, risk of harm to another). If the individual satisfies criterion A but not B, there is no paraphilic disorder, but paraphilia. Therefore, this new concept accepts atypical sexual interests, as long as it is consensual, as not being pathological. This implies that the individuals involved are not incapable of giving their consent (such as children or mentally disabled persons, for example). The simple presentation of these new concepts and diagnostic criteria has raised doubts and heated discussions in the academic field, which will have repercussions in clinical practice. Finally, they reflect a revolution in the form in which North-American specialists understand sexual behavior and function today. We await with curiosity for what ICD-11 will propose.
